# Carrying Asymmetric Loads While Walking on a Treadmill Interferes with Lower Limb Coordination

**DOI:** 10.3390/ijerph18094549

**Published:** 2021-04-25

**Authors:** Junsig Wang, Mitchell L. Stephenson, Chris J. Hass, Christopher M. Janelle, Mark D. Tillman

**Affiliations:** 1 Department of Orthopaedic Surgery, University of Arkansas for Medical Science, Little Rock, AR 72205, USA; 2 Department of Health and Human Performance, University of Montana Western, Dillon, MT 59725, USA; mitchell.stephenson@umwestern.edu; 3 Department of Applied Physiology and Kinesiology, University of Florida, Gainesville, FL 32611, USA; cjhass@aa.ufl.edu (C.J.H.); cjmj@hhp.ufl.edu (C.M.J.); 4 Brooks Rehabilitation College of Healthcare Sciences, Jacksonville University, Jacksonville, FL 32211, USA; mtillma3@ju.edu

**Keywords:** asymmetric load, gait kinematics, continuous relative phase, treadmill walking

## Abstract

The purpose of this study was to investigate the effect of different load carriage modes on coordinative patterns in the lower extremities during walking. Twenty-five university students walked on a treadmill at their preferred pace under three different load conditions: symmetric load (5% of body mass in messenger bags on each shoulder hanging vertically and against the hips), asymmetric load 1 (10% of body mass in a messenger bag on one shoulder hanging vertically against the ipsilateral hip), and asymmetric load 2 (10% of body mass in a messenger bag on one shoulder with the bag draped across the trunk to the contralateral hip). Altered thigh-shank and shank-foot couplings were found for the loaded side during the stance of gait when comparing the asymmetric 1 and 2 to the symmetric load. In addition, thigh-thigh coupling was changed during gait when comparing the asymmetric load 2 and symmetric load. However, we did not find any significant differences in intralimb and interlimb couplings between the two different asymmetric load conditions. The results suggest potential benefits when carrying symmetrical loads in order to decrease abnormal limb coordination in daily activities. Thus, it may be advisable to distribute load more symmetrically to avoid abnormal gait.

## 1. Introduction

People frequently carry loads using bags with shoulder straps, permitting them to transport a variety of items and still have their hands free. Single strap messenger bags have become increasingly popular, leading to a large proportion of individuals carrying their loads asymmetrically. Walking while carrying an asymmetric load results in shorter stride length, faster cadence, and shorter step width than an unloaded condition [1,2]. A recent study reported decreased gait stability during asymmetric load carriage than bilateral load carriage, but no difference in cadence, stride length, and step width between unilateral and bilateral load carriage [3]. These studies did not find a significant difference between unilateral and bilateral load carriage in temporo-spatial gait parameters. However, the previous investigation of load carriage has been limited to the simple gait variables and thus there is a need to assess gait mechanics with a more advanced approach for better understanding of lower limb adaptation.

Limb coordination refers to how two adjacent segments interact or couple together temporally and spatially. In gait, limb coordination is crucially important and must be altered according to the demands of varying external circumstances [4,5,6,7]. To adapt to the demands of various environments, specific coordinative patterns occur both within limb (intralimb) segments and between limbs (interlimb), which may be considered an important predictor for potential trip/fall-related injuries. A previous study assessed lower limb coordination when carrying an asymmetric load in a hand-held bag and found that 3 kg and 8 kg asymmetric loads did not change lower limb coordination [8]. However, few studies have been performed to investigate adaptive limb mechanisms in the lower extremity when carrying asymmetric loads. Indeed, load carriage can be a common reason for fall-related injuries in workplace tasks [9]. A previous study also indicated that carrying about 10% body mass loads could impact college student pedestrian safety [10]. Thus, understanding of lower limb mechanisms with different methods of carrying loads may help guide effective gait training programs aimed at improving balance control (fall prevention) and safe pedestrian behavior during asymmetric load carriage, which may be of value for public health.

Researchers have used different nonlinear dynamic techniques to investigate variability in human movement based on the dynamical systems theory [11]. One prevalent dynamical system analysis for studying coordination of segmental movement can be evaluated through the continuous relative phase (CRP). This measure has been used to quantify the coordination between different body segments in several activities by recreating it as a dynamic system and studying its stabilizing features during the entire movement cycle [12]. Therefore, the relative phases of several interacting segments can be measured to quantify segmental coordination and evaluate movement patterns.

Our aim, therefore, was to evaluate gait kinematics and lower extremity limb coordination in response to different load conditions induced through the manipulation of the amount and location of loads carried. We hypothesized that intralimb coordination (thigh–shank and shank–foot) and interlimb coordination (thigh–thigh, shank–shank, and foot–foot) for symmetric load carriage would be closer to the unloaded condition than asymmetric load carriage.

## 2. Materials and Methods

Twenty-five healthy college students with an age range of 18 to 30 years (12 males and 13 females; age 21.6 ± 3.6 years; height 170.9 ± 8.5 cm; mass 67.2 ± 12.5 kg) participated in this research. All were free of any pathology that would prevent them from walking on a treadmill. Prior to participating in the study, each participant read and signed an informed consent form approved by the university’s institutional review board.

**Figure 1:**
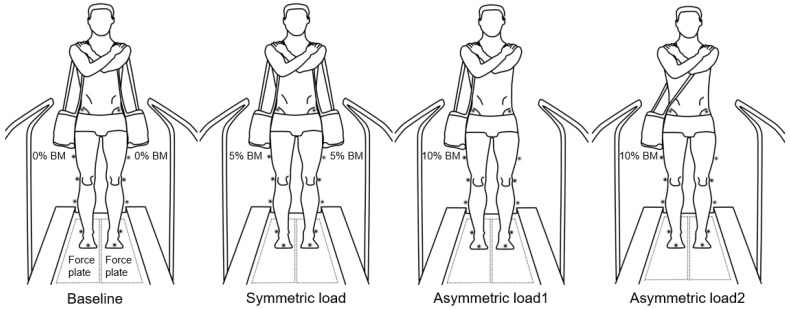
Illustration of four different load conditions. Baseline (no load with one messenger bag on each shoulder hanging vertically down to the hips) symmetric load (5% of body mass in messenger bags on each shoulder hanging vertically), asymmetric load 1 (10% of body mass messenger bag on one shoulder hanging vertically against ipsilateral hip), and asymmetric load 2 (10% of body mass messenger bag on one shoulder with the bag draped across the trunk to contralateral hip).

Two single strap bags were utilized to create three different experimental load conditions and one baseline condition: Two single strap bags (baseline), one on each shoulder and hanging down vertically (0% of body mass (BM), two 5% BM single strap bags with one on the right shoulder and one on the left (symmetric load) and a 10% BM single strap bags in different positions (asymmetric load 1 and 2, Figure 1). The average 10% body mass load carried was 6.7 ± 1.3 kg. Two empty bags were carried during the baseline condition in order to isolate the direct effect of amount of load and load asymmetry from the effect of simply wearing the bags. Participants walked on a treadmill in all conditions with arms crossed, hands on each opposite shoulder (Figure 1). Previous studies using loads of this magnitude (10–20% BM) have observed altered locomotor behavior [13,14,15,16]. It was reported that average school bag mass was close to 10% BM [17]. Additionally, a load carriage recommendation for a school bag includes no more than 10% BM load [18]. Furthermore, the messenger bags were positioned approximately near the anterior superior iliac spines (ASIS) kinematic markers across the conditions to reduce marker obstruction during data collection. Twenty-three participants were right-handed and preferred to carry a messenger bag on the right shoulder, while the two left-handed individuals preferred to carry the bag on left shoulder. Though both sides were loaded during the symmetric load, the ‘loaded’ and ‘unloaded’ labels in the remaining portions of this study refer to the sides which are loaded or unloaded during the asymmetric load 1 and 2, respectively.

A motion analysis system with seven high-resolution cameras (Vicon Nexus, Oxford, UK), was used to collect three-dimensional kinematic data (at 120 Hz) during each testing condition. The treadmill was instrumented with two force platforms that allowed for measuring continuous ground reaction force data (at 1200 Hz) during gait (Bertec Corporation, Columbus, OH, USA). Force data were used for identifying gait events (toe-off and heel strike).

Sixteen retro-reflective markers were placed on the lower extremity over bony landmarks following the Vicon Plug-in-Gait (lower body) marker system. The marker set included bilateral great toes, heels, lateral malleoli, lateral calves, lateral knee joint lines, lateral thighs, ASIS, and posterior superior iliac spines (PSIS). All participants were asked to walk on a treadmill at their preferred pace for five minutes in each condition. To determine this pace, the treadmill belt speed was initiated at 0.5 m/s and was gradually increased in increments of 0.1 m/s until the participant signaled that their preferred speed had been reached. This speed was then held constant during data collection. Testing order of the conditions was randomly assigned. The last minute of the five minutes in each condition was recorded and extracted for analysis. Thus, approximately 50 strides in each condition were recorded. Marker trajectories were filtered using a fourth-order Butterworth filter with a low pass cutoff frequency of 10 Hz. Gait cycles were divided into stance and swing phases identified from the force data. 

**Figure 2:**
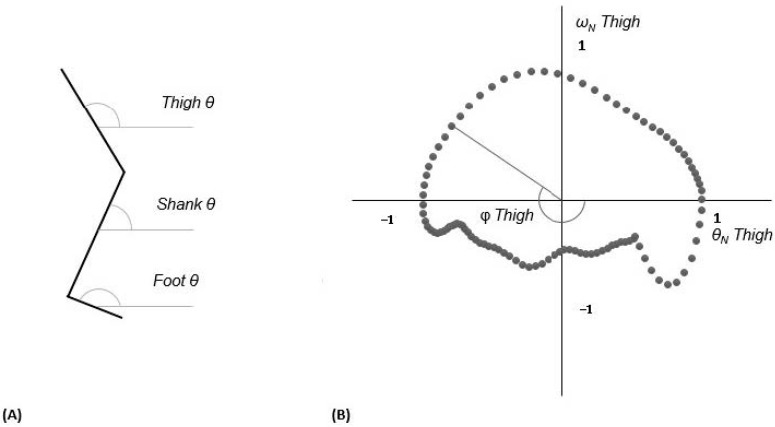
(**A**) Illustration of each segmental angle: thigh, shank, and foot angles in sagittal plane. (**B**) Phase plot illustrating phase angle based on angular displacement versus angular velocity over one gait cycle. Calculation of phase angle (φ) of thigh was obtained from arctangent function of angular velocity (ω)/angular displacement (θ).

Three segmental angles (thigh, shank, and foot) were exported from the Vicon system. The segmental angles were temporally normalized to 100% of the gait cycle (101 data points). Angular velocities were calculated in the sagittal plane utilizing the first central difference method [19]. Continuous relative phase (CRP) analyses were performed to identify intralimb coordination between the thigh and the shank and between the shank and the foot of each leg, as well as the interlimb coordination between each segment of each leg. The time-normalized angles and angular velocities were used for the CRP calculation. These data were then used to calculate phase angles from a phase plot (Figure 2), using the arctangent of angular velocity/angular displacement at each data point. Prior to calculation for CRP, each segment angle was normalized for each trial using Equation (1) [7,20]. CRPs in different frequency signals can be understandable results and prevent distorted raw data through this normalization [21].

(1) $$\mathrm{angle}\;:\theta_{N_{i}}=\frac{2*[\theta_{i}-(\theta_{\max}+\theta_{\min})]}{\theta_{\max}-\theta_{\min}}$$

θ: angle; $\theta_{N}$: normalized angle; $\theta_{\max}$: maximum angle within one gait cycle; $\theta_{\min}$: minimum angle within one gait cycle; i: data point (1/100 s).

Also, angular velocity was normalized using the following equation:

(2) $$\mathrm{angula}\;\mathrm{velocity}\;:\omega_{N_{i}}=\frac{\omega_{i}}{\max\left\{\max\left(\omega_{i}\right),\min\left(-\omega_{i}\right)\right\}}$$

ω: angular velocity (thigh, shank, and foot); $\omega_{N}$: normalized angular velocity; max($\omega_{i}$): maximuam angular velocity within one gait cycle; min(–$\omega_{i}$): minimum angular velcity within one gait cycle; i: data point (1/100 s).

The phase angles (φ) were obtained by calculating four-quadrant arctangent of the ratio of angular velocity by angular position:

(3) $$\varphi_{i}=\tan^{-1}\left[\frac{\theta_{N_{i}}}{\omega_{N_{i}}}\right]$$

φ: phase angle; i: data point (1/100 s).

Then the CRP was calculated by subtracting the phase angle of the proximal segment from that of the distal segment for a specific point during the gait cycle [7,20,22].

(4) $$\mathrm{CRP}=\varphi_{\mathrm{proximal}}-\varphi_{\mathrm{distal}}$$

When the CRP is near 0°, the respective segments are in-phase, while 180°of the CRP indicates that both segments are out-of-phase [11]. Positive relative values indicate that the distal segment is ahead of the proximal segment, and negative values indicate that the proximal segment is ahead in phase space [11]. For interlimb coupling, CRP was calculated by taking the difference between the phase angles of both segments for each data point. The interlimb couplings were the thigh–thigh, shank–shank, and foot–foot. Coordination patterns were quantified utilizing cross-correlation coefficient (CCC) and root-mean-square (RMS) techniques. CCC was assessed by comparing the average CRP in each load condition to the average CRP in the baseline condition for interlimb and intralimb couplings. RMS difference was also evaluated by comparing the average CRP in each load condition to the average CRP in the baseline condition. While the CCC measure indicates changes in the spatio-temporal evolution of CRP patterns, RMS measures provide information about the magnitude differences in relative phase between the patterns [7].

Statistical analyses were performed using SPSS^®^ (version 20; SPSS Inc., Chicago, IL, USA). Repeated measures analysis of variance (ANOVA) was performed on the 14 limb coordination parameters: seven RMSs and seven CCCs (thigh-shank in the unloaded side, thigh–shank in the loaded side, shank–foot in the unloaded side, shank foot in the loaded side, thigh-thigh, shank-shank, and foot-foot). Significance was again set at an a priori 0.05 via a Bonferroni correction of fourteen (the number of dependent variables) for limb coordination parameters. The fourteen variables were not normally distributed by Shapiro–Wilk tests of normality. Therefore, when significant main effects were detected, Wilcoxon signed-rank tests were performed at a 0.05 level. 

## 3. Results

RMS differences for thigh-shank coupling during the stance phase in the loaded side were observed. The following Wilcoxon signed-rank test revealed that thigh-shank coupling during the asymmetric load 1 and 2 were greater than the symmetric load (*p* = 0.007 & *p* < 0.001; Table 1). For shank-foot coupling, significant differences in RMS were observed on the loaded side during stance. During the stance phase, RMS increased during the asymmetric load 1 and 2 in the loaded side compared to the symmetric load (*p* = 0.011 & *p* = 0.001, respectively; Table 1). No statistically significant CCC effects for thigh-shank and shank-foot coupling for either limb were detected (Table 1). All CCC values for intralimb coupling were close to 1. Additional graphical analyses (with mean ensemble curves of CPR) were performed and thus the interpretation was included in the discussion. 

Interlimb coordination was examined via thigh–thigh, shank–shank, and foot–foot couplings. No effects on RMS changes for interlimb coupling were observed (Table 2). However, CCC in thigh–thigh pairing varied across the conditions. CCC for thigh–thigh coupling during the asymmetric load 2 was significantly decreased compared to the symmetric load (*p* = 0.01; Table 2). Also, no effect on CCC in shank–shank and foot–foot coupling was displayed.

## 4. Discussion

We investigated coordinative lower extremity mechanisms in response to different loading conditions during treadmill walking. As hypothesized, coordination was altered during unilateral load carriage. Specifically, increased RMS values for intralimb and interlimb coordination were found during asymmetrical load carriage. Also, a decreased CCC value for interlimb (thigh–thigh) coordination was observed during the asymmetrical load carriage.

The RMS difference in thigh–shank coupling for the loaded limb during stance was increased for the asymmetric load 1 (10% BM on one shoulder hanging vertically against ipsilateral hip) and the asymmetric load 2 (10% BM messenger bag on one shoulder with the bag draped across the trunk to the contralateral hip) compared to the symmetric load (5% BM messenger bags on each shoulder hanging vertically). Similar tendencies for RMS differences between no load and unilateral leg load conditions have been detected in previous research [7]. We observed no RMS changes on the unloaded side in thigh–shank couplings. In part, the findings of the previous study may contrast our results due to different methods of carrying loads. Specifically, Haddad et al., (2006) utilized a custom-made leg loading device that was placed 2.5 cm above the lateral malleolus as opposed to our upper body load carriage conditions that better reflect common load carriage techniques [7]. 

**Figure 3:**
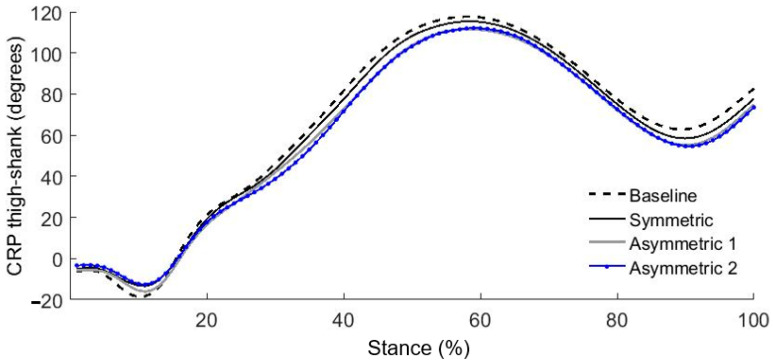
Mean continuous relative phase (CRP) curves in thigh-shank on the loaded side during stance phase for baseline (no load) and each experimental condition (*n* = 25).

Complete CRP curves provide information regarding how in phase or out of phase two segments are during the entire stance phase. For example, the CRP pattern of thigh-shank coupling in the loaded side for the asymmetric load 1 and 2 were less out-of-phase in mid to late stance phase (40–60% and 80–100%) than the symmetric load (Figure 3). Therefore, these alterations in thigh-shank coupling produced greater RMS values that indicate restricted thigh-shank coupling in the loaded side during the unilateral load carriage on one shoulder. Less out-of-phase thigh-shank patterns have been seen in hemiparetic gait [20]. Similar patterns of the less out-of-phase thigh–shank coupling have also been observed when an orthotic knee constraint was applied in healthy participants [20]. Ultimately, the loss of the thigh lead over the shank during stance phase may restrict knee flexion during gait, leading to less than optimal performance. Thus, less thigh lead over shank may violate stability in ipsilateral limb motion during the gait.

**Figure 4:**
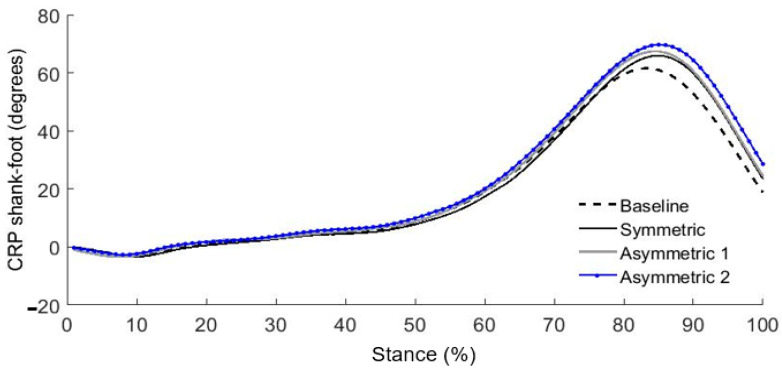
Mean continuous relative phase (CRP) curves in shank-foot on the loaded side during stance phase for baseline (no load) and each experimental condition (*n* = 25).

For shank–foot coupling, we also found significant RMS differences only on the loaded side during the stance phase. Again, these RMS differences provide only partial information. Further graphical analysis (Figure 4) revealed that the RMS differences for the asymmetric load 1 and 2 result from more out-of-phase coupling compared to the symmetric load. The CRP curves for the asymmetric load 1 and 2 during late stance phase (60–100%) show more out-of-phase movement compared to the symmetric load. These two asymmetrical loading conditions contributed to intensified out-of-phase shank–foot coupling on the loaded side during late stance (Figure 4). We also noted altered coordinative patterns in thigh–shank and shank–foot couplings only on the loaded side.

The functional purpose of these alterations on the loaded side remains unclear but may be related to function of two joint muscles behaving differently in this loaded state as well as altering individual joint range of motion. Previously, researchers reported a decrease in peak ankle dorsiflexion on the loaded side during braking phase when carrying asymmetrical load, which may lead to increased plantarflexion during propulsion [23]. Furthermore, plantar flexors and knee flexor muscles may be associated with this adaptation during propulsive period (mid-to-late stance). However, a more comprehensive interpretation might be completed by electromyography and kinetic analyses in a future study.

Another interesting finding in the current study is that CRP curves in thigh–shank and shank–foot display substantially different adaptations in response to asymmetric loading. Thigh-shank couplings on the loaded side during stance were less out-of-phase, while shank–foot coupling showed a more out-of-phase pattern on the loaded side during stance with an asymmetrical loading compared to symmetric loading. Thus, it is possible that increased out-of-phase shank–foot coupling may be related to compensatory adaptation in both knee and ankle joints [24,25,26]. For example, additional loads concentrated on the loaded limb stance may result in relatively in-phase coordination for thigh–shank and out-of-phase for shank–foot during mid-late stance phase (plantar-flexion phase). In-phasing thigh–shank was observed in a stiff limb of hemiparetic gait [27]. Holt et al., (2003) reported that load carriage resulted in increased stiffness about knee, hypothetically suggesting a higher demand for muscle coactivity to increase stiffness in response to carried loads [28]. Moreover, contribution of ankle plantarflexion during the late stance phase might be increased due to increased stiffness about knee. Extensor ankle torque was increased while the extensor knee torque was decreased with backpack (15–30% BM) after a 40 min walk [25]. Also, pregnant women exhibited increased extensor ankle moment and power to compensate for change in body mass [26]. Therefore, additional body mass may lead to the compensatory mechanism in both knee and ankle joints in this acute evaluation. Furthermore, these alterations between two joints on the loaded limb may intensify asymmetry between limbs.

The effect of asymmetric load carriage was also observed for interlimb coordination. As mentioned before, CCC value in thigh–thigh coupling was decreased when carrying a messenger bag on one shoulder (the asymmetric load 2), indicating a greater difference in coordination compared to carrying two messenger bags (one on each shoulder). In the current study, increased asymmetry in interlimb coordination was found only for thigh-thigh coupling in terms of CCC measure. In general, smoothness and symmetry in gait are regarded as ‘normal walking’. Asymmetry during gait, as observed here, may be considered potentially injurious as an asymmetrical gait pattern could lead to significant stresses in the lower extremity [29], higher energy cost [30], and inefficient mechanisms in the lower body [31].

Adaptations in limb coordination during asymmetrical load carriage should be further investigated, as they may be indicative of the possibility of acute and/or chronic joint injury and pain. Alteration in the loaded limb when carrying an asymmetric load may result in restricted knee motion and increased stiffness about knee, relying on the ankle joint during the stance phase. Therefore, it remains to be seen if these repetitive and long-term changes may result in higher risk of knee injuries [32]. If this is the case, it may be beneficial to prevent people from carrying asymmetrical load when possible.

There are several limitations in the current study beyond the aforementioned considerations. One of the limitations is that arm movement was suppressed across the three load conditions. However, arm movements are indispensable parts of human locomotion that play an important role in balance and coordination [33,34]. The participants tested here selected a slower speed (0.8 m/s) than normal walking velocity as their preferred walking speed. The slower gait speed during treadmill walking can impact gait mechanics [35]. Another limitation is no assessment of the trunk and pelvis adaptations relative to the lower limbs. Changes in upper body mechanisms could be an important adjustment to preserve dynamic balance during these conditions. Therefore, it is suggested that following investigations should focus on the coordinated relationship between the torso and lower extremity. Finally, the evaluation of limb coordination in other planes (frontal and transverse) may be required to complete a holistic analysis of the subsequent lower limb adaptations.

## 5. Conclusions

Our findings suggest a variety of adaptations in intralimb and interlimb coordination in response to symmetric and asymmetric load carriage. Changes in intralimb and interlimb coordination were observed for the asymmetrical loading conditions compared to symmetric loading condition. However, the two different asymmetrical conditions did not show any difference in interlimb and intralimb couplings. We observed adaptive patterns in thigh–shank, shank–foot, and thigh–thigh coordination. Based on our findings, we suggest potential benefits when carrying symmetrical loads in order to decrease abnormal limb coordination in daily activities. These alterations may provide researchers with preliminary knowledge concerning diverse gait adaptations caused by external constraints. Practically speaking, the results of this investigation continue to support the premise that carrying loads at 10% of body mass may potentially cause increased injury risk, notably in asymmetric carriage methodologies. As modifications in movement coordination may undermine balance control, we recommend caution in carrying asymmetric loads, particularly in populations that may be at increased trip and fall risk due to other morbidities.

## Figures and Tables

**Table 1:** Root mean square (RMS) difference and cross-correlation coefficient (CCC) for thigh-shank and shank-foot couplings (intralimb coordination) during stance for the loaded side and unloaded side. ^a^ *p* < 0.05 vs. symmetric load.

	Symmetric	Asymmetric 1	Asymmetric 2	Main Effect
	Mean (*SD*)	Mean (*SD*)	Mean (*SD*)	*p*-Value
Thigh–shank RMS for loaded	5.93	9.09 ^a^	10.01 ^a^	<0.001
	(2.27)	(5.82)	(5.06)	
Shank–foot RMS for loaded	3.53	5.01 ^a^	5.96 ^a^	<0.001
	(1.98)	(2.38)	(2.85)	
Thigh–shank RMS for unloaded	8.38	11.34	10.15	1.000
	(3.34)	(7.71)	(6.50)	
Shank–foot RMS for unloaded	4.73	5.73	5.47	1.000
	(2.25)	(2.91)	(3.40)	
Thigh–shank CCC for loaded	0.996	0.994	0.992	0.392
	(0.005)	(0.007)	(0.007)	
Shank–foot CCC for loaded	0.990	0.990	0.983	0.558
	(0.012)	(0.007)	(0.017)	
Thigh–shank CCC for unloaded	0.995	0.991	0.993	1.000
	(0.004)	(0.011)	(0.007)	
Shank–thigh CCC for unloaded	0.990	0.986	0.990	1.000
	(0.013)	(0.015)	(0.011)	

**Table 2:** Root mean square (RMS) difference and cross-correlation coefficient (CCC) for thigh-thigh, shank-shank, foot-foot couplings (interlimb coordination) over a gait cycle. ^a^ *p* < 0.05 vs. symmetric load.

	Symmetric	Asymmetric 1	Asymmetric 2	Main Effect
	Mean (*SD*)	Mean (*SD*)	Mean (*SD*)	*p*-Value
Thigh–thigh RMS	3.00	4.02	4.19	0.182
	(1.02)	(2.13)	(1.90)	
Shank–shank RMS	2.89	3.17	3.60	1.000
	(1.58)	(1.74)	(2.23)	
Foot–foot RMS	3.57	3.61	4.04	1.000
	(2.45)	(1.16)	(2.24)	
Thigh–thigh CCC	0.976	0.964	0.957 ^a^	0.042
	(0.011)	(0.032)	(0.027)	
Shank–shank CCC	0.976	0.973	0.970	1.000
	(0.015)	(0.019)	(0.030)	
Foot–foot CCC	0.971	0.970	0.966	1.000
	(0.027)	(0.017)	(0.034)	

## Data Availability

The data presented in this study are available on request from the corresponding author.
